# Monitoring and Evaluation of Emotion Regulation by Aerobic Exercise and Motor Imagery Based on Functional Near-Infrared Spectroscopy

**DOI:** 10.3389/fncom.2021.759360

**Published:** 2021-10-27

**Authors:** Peng Ding, Fawang Wang, Siyu Li, Wei Zhang, Hongquan Li, Zhuangfei Chen, Lei Zhao, Anmin Gong, Yunfa Fu

**Affiliations:** ^1^Faculty of Information Engineering and Automation, Kunming University of Science and Technology, Kunming, China; ^2^Brain Cognition and Brain-Computer Intelligence Integration Innovation Group, Kunming University of Science and Technology, Kunming, China; ^3^Brain Science and Visual Cognition Research Center, Medical School of Kunming University of Science and Technology, Kunming, China; ^4^School of Rehabilitation, Kunming Medical University, Kunming, China; ^5^Faculty of Science, Kunming University of Science and Technology, Kunming, China; ^6^Information Engineering College, Engineering University of People's Armed Police, Xi'an, China

**Keywords:** functional near-infrared spectroscopy, aerobic exercise, emotion regulation, motor imagery, state-trait anxiety inventory, profile of mood states

## Abstract

**Objective:** We sought to effectively alleviate the emotion of individuals with anxiety and depression, and explore the effects of aerobic exercise on their emotion regulation. Functional near-infrared spectroscopy (fNIRS) brain imaging technology is used to monitor and evaluate the process of aerobic exercise and imagination that regulates emotion.

**Approach:**Thirty participants were scored by the state-trait anxiety inventory (STAI) and profile of mood states (POMS), and fNIRS images were collected before, after, and during aerobic exercise and motor imagery. Then, the oxygenated hemoglobin (HbO), deoxygenated hemoglobin (HbR), and total hemoglobin (HbT) concentrations and their average value were calculated, and the ratio of HbO concentration in the left and right frontal lobes was determined. Spearman's correlation coefficient was used to calculate the correlation between variations in the average scores of the two scales and in blood oxygen concentrations.

**Results:** In comparison with motor imagery, STAI, and POMS scores decreased after 20 min of aerobic exercise. The prefrontal cortex had asymmetry and laterality (with the left side being dominant in emotion regulation). The increase in hemoglobin concentration recorded by fNIRS was negatively correlated with STAI and POMS scores. Aerobic exercise has a good effect on emotion regulation.

**Significance:**The study showed that portable fNIRS could be effectively used for monitoring and evaluating emotion regulation by aerobic exercise. This study is expected to provide ideas for constructing fNIRS-based online real-time monitoring and evaluation of emotion regulation by aerobic exercise.

## Introduction

The rapid development of society has placed a certain amount of pressure on individuals or groups, which can trigger different degrees of anxiety or depression. If individuals do not pay attention to emotion regulation, physical, and mental illnesses may occur, with severe ones capable of leading to mental dysfunction or adverse social events (Veerapa et al., 2020). Aerobic exercise is one option to improve the mood and promote the generation of positive emotions (Brush et al., 2020) that can be used by people with severe anxiety for emotion regulation (Tempest and Parfitt, 2013). However, there is currently a lack of monitoring and evaluation of aerobic exercise in mood regulation. Meanwhile, the pleasant, comfortable, or energetic experience of motor imagery may also further promote the regulation of emotion (Tempest and Parfitt, 2013), but its regulatory effect on motion is still unclear. Therefore, in the study, functional near-infrared spectroscopy (fNIRS) brain imaging was used to monitor and evaluate the effect of aerobic exercise and motor imagery on emotion regulation (Jiang et al., 2017; Veerapa et al., 2020).

Improving the emotional health of people with anxiety is very important. We hypothesize that corresponding aerobic exercise imagination may also help to improve the mood of individuals with anxiety, just as the memory of a good experience can enhance their mood. Motor imagery based on aerobic exercise was designed to verify our hypothesis. In general, aerobic exercise of an individual is familiar, easy, and can be carried out naturally, habitually, and automatically. In this context, the brain does not need to recruit too many nervous system resources. Therefore, individuals can allocate certain psychological resources to carry out aerobic exercise imagination when they perform aerobic exercise automatically (Tempest and Parfitt, 2013). The present study used fNIRS to monitor and evaluate emotion regulation by aerobic exercise and motor imagery.

Sports medicine shows that aerobic exercise is a type of physical exercise that can involve the full exchange of oxygen to achieve physiological balance. At the same time, the heart rate needs to reach 150 bpm before aerobic exercise; therefore, aerobic exercise generally includes moderate- or high-intensity activities (60–80% of the maximum heart rate), such as jogging, walking, fast running, cycling, swimming, or rope skipping. These exercises are considered aerobic exercise, which is conducive to the health of the body (Cheng, 2007). Practice and sports medicine studies also have suggested that aerobic exercise can regulate emotion, alter mood, improve negative emotions, and promote the production of positive emotions (Ekkekakis et al., 2013; Tempest et al., 2014; Bernstein and McNally, 2015; Bernstein and Mcnally, 2017; Edwards et al., 2017, 2018; Brush et al., 2020). However, the changes of blood oxygen metabolism in brain tissue under aerobic exercise and the relationship between these changes and emotional state need to be further discussed.

In comparison with electroencephalography (EEG), fNIRS is less sensitive to motion artifacts, has a good ecological effect, and can tolerate a certain degree of exercise interference (Sitaram et al., 2007; Cui et al., 2011; Naseer and Hong, 2013). Moreover, fNIRS can measure the blood oxygen metabolism (HbO and HbR) of brain tissue during aerobic exercise, while EEG measures the discharge activities of central neurons. The spatial resolution and spatial positioning accuracy of fNIRS are better than those of EEG. fNIRS is also non-invasive and portable. Thus, relative to EEG, fNIRS may be more suitable for monitoring and evaluating the emotion-regulation effects of aerobic exercise. In addition, as compared with fNIRS, functional magnetic resonance imaging and magnetoencephalography are bulky, not portable, and expensive, and are not suitable for monitoring and evaluating brain activity during aerobic exercise emotion regulation (Weiskopf et al., 2004; Goldin et al., 2013).

Some research has examined the influence of aerobic exercise on changes in the HbO concentration as measured by fNIRS (Chen et al., 2017), but few investigators have evaluated its impact on changes in HbR and HbT, as measured by fNIRS. In the present study, three characteristics of fNIRS (HbO, HbR, and HbT) were extracted to evaluate the concentration changes that occurred between before and after exercise and the ratio of HbO concentration changes between the left and right prefrontal lobes before and after aerobic exercise, and motor imagery was calculated to investigate the lateralization of the activation of brain regions. Blackhart et al. used a questionnaire for pre- and post-test assessments to verify that the degree of left frontal EEG can predict the symptoms of anxiety and depression (Blackhart et al., 2006), while Smith et al. used a model to calculate the risk of anxiety and depression and revealed the correlation between the degree of left frontal EEG and said risk (Smit et al., 2007), which also supported the conclusions of Blackhart et al. At the same time, patients with anxiety and depression also exhibit decreased left frontal lobe activity. Therefore, the degree of left lateralization of frontal EEG can be used as an indicator of anxiety and depression to a certain extent and to predict the development of symptoms. However, fNIRS has not been used to observe frontal lobe asymmetry nor has it been applied to detect the effects of aerobic exercise on emotion regulation. Therefore, we chose to use fNIRS to observe these two brain regions and analyze the relationship between the changes in neural mechanisms and emotion regulation in these two brain regions to confirm the hypothesis of this experiment, which is as follows: fNIRS can be used as a means of monitoring and evaluating emotion regulation. We present a three-dimensional topographic map of the dynamic changes in HbO concentration that occur during aerobic exercise. In addition, previous studies have used either the profile of mood states (POMS) (Chen et al., 2017) or the state-trait anxiety inventory (STAI) (Chen et al., 2019; Clemente-Suárez, 2020) for assessing the emotion regulation of individuals with state-trait anxiety. To more comprehensively evaluate the effects of emotion regulation by aerobic exercise, POMS and STAI were used in this study, and an ANOVA was used to analyze aerobic exercise and motor imagery, focusing on the significance of changes in the two scores between before and after aerobic exercise.

In addition to the above-mentioned assessment of emotion regulation by aerobic exercise, few people have studied whether motor imagery can regulate emotion, and especially few people have monitored and evaluated it using fNIRS. Francesca et al. showed that motor imagery can promote or inhibit related neural activities and then regulate individual anxiety (Fardo et al., 2015). Shafir et al. also reported that individuals can regulate their own emotions by imagining aerobic exercise or by means conducive to regulating their anxiety (Tal, 2016). In addition, until now, few people have explored the correlation between STAI and POMS scores and the changes in HbO, HbR, and HbT concentrations based on fNIRS. In this study, Spearman's correlation coefficient was used to analyze the correlation between STAI and POMS scores and changes in the HbO, HbR, and HbT concentrations. The emotion subscale has a certain participantivity in the evaluation of emotional changes (Knapen et al., 2009; Szabo et al., 2015; Subramaniapillai et al., 2016; Bernstein and Mcnally, 2018). In addition to using STAI and the POMS emotion subscale to monitor and evaluate emotional changes, fNIRS technology was introduced to monitor and evaluate changes in cerebral blood oxygen metabolism before, during, and after aerobic exercise, which is expected to improve the objectivity of emotion monitoring and evaluation. The present study is expected to provide ideas for developing fNIRS-based online real-time monitoring and evaluation of emotion regulation by aerobic exercise and motor imagery, which can be used to monitor and evaluate individual state-trait anxiety and mood states.

## Materials and Methods

### Research Scheme

**Figure 1:**
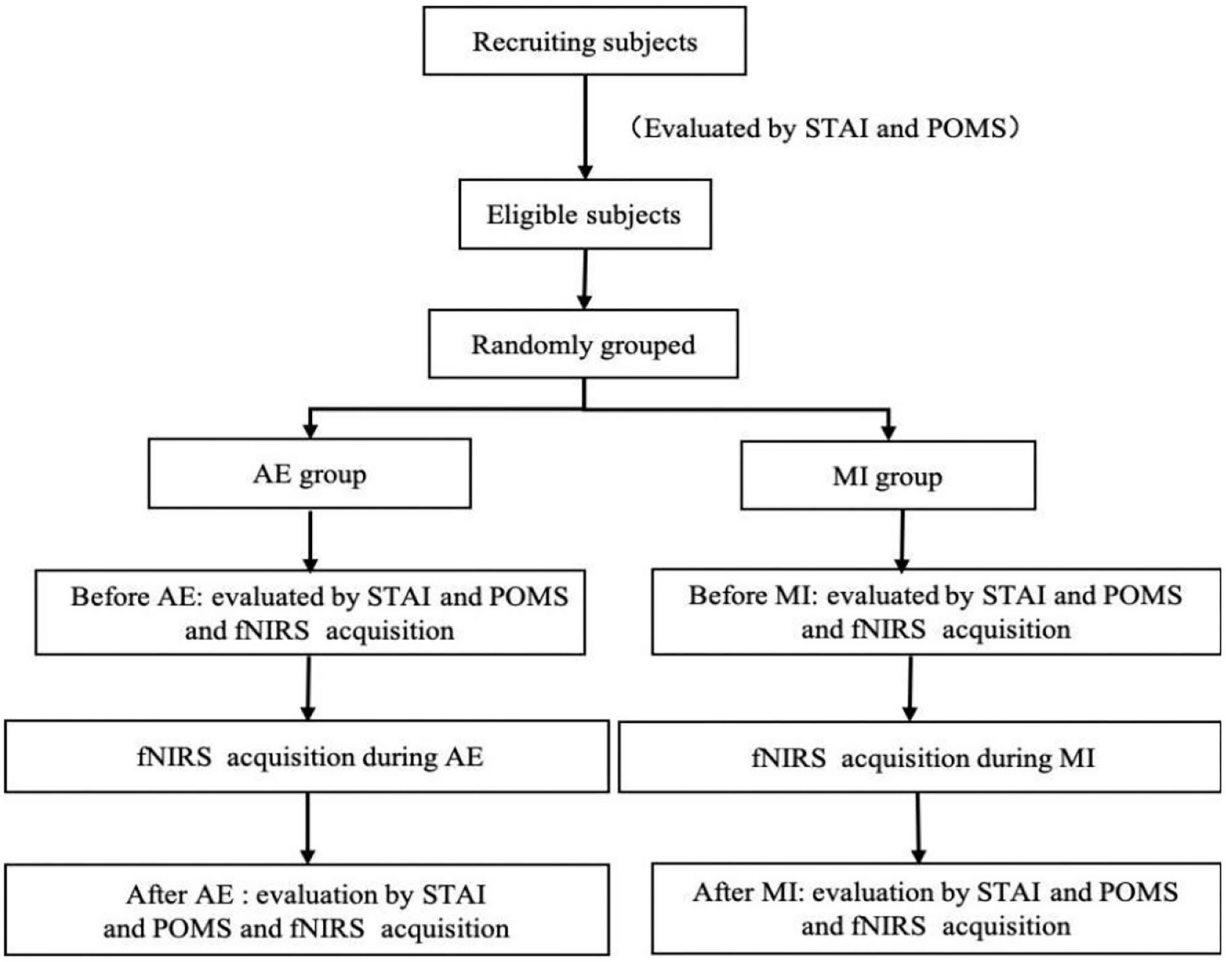
Schematic diagram of the study process.

Figure 1 shows a schematic diagram of the scheme of this study, which will be described in detail henceforth. In this study, eligible subjects were randomly divided into an aerobic exercise group and exercise imagination group, and then STAI and POMS were evaluated, and fNIRS was collected before and after aerobic exercise and exercise imagination tasks. Perform fNIRS acquisition during the mission.

### Study Participants

A total of 30 participants (21 men, average age: 23 ± 2.0 years, age range: 21–26 years; nine women, average age: 23 ± 2.0 years) were asked to complete STAI and POMS to evaluate whether they met the inclusion criteria (STAI score of 40–59 points and POMS score of 110–140 points). The selected participants were divided into two groups (*n* = 15 each), an aerobic exercise group (S1–S15) and a motor imagery group (S16–S30), according to their height, body shape, sex, age, and other factors; there was no significant difference in these factors between the two groups. All of the participants were undergraduates or graduate students, were right-handed, had no history of mental, neurological, or musculoskeletal disease or drug abuse, had normal or corrected vision, and had no color blindness. All of the participants signed the experimental informed consent form, and this study was approved by the medical ethics committee of the Kunming University of Science and Technology School of Medicine.

### Participant Training

The aerobic exercise group performed warm-up exercises for 5 min, and then aerobic exercise (Perini et al., 2016) (refer to section Experimental Equipment and Data Collection for exercise requirements) using a horizontal magnetic bicycle (the resistance was adjusted to four levels of medium resistance) for 1 min to adapt to the machine, then rested for 3 min (walking and relaxing), after which point a computer voice prompted emotion regulation by aerobic exercise. The experiment began and the participants performed aerobic exercise for 20 min until the end of the voice prompt experiment. Participants in the motor imagery group first performed the aerobic exercise with the horizontal magnetic bicycle (resistance adjusted to four levels of medium resistance) for 1 min to experience the actual aerobic exercise process and then were asked to rehearse or feel the aerobic exercise process from the first-person perspective—but no actual movement occurred (i.e., kinesthetic imagery) (Proske and Gandevia, 2018). Specifically, they repeated the actual moving process for 1 min and, at the same time, used motor imagery to evoke the pleasure or comfort brought by the movement, then rested for 3 min before the computer voice prompted the motor imagery experiment to begin and the participants performed the motor imagery experiment for a continued 20 min until the end of the voice prompt experiment. Before and after the experiment, the participants were required to fill in the STAI and POMS questionnaires.

Aerobic exercise involves moderate- or high-intensity exercise, so recumbent cycling can be divided into eight levels of intensity, with level 4 representing moderate-intensity exercise; thus, it is necessary for this study to ensure that exercise was performed above this level. During the experiment, the heart rate and exercise time of the participants were recorded by the recumbent cycle. To accurately grasp the HR_max_ of the study participants, before the beginning of the experiment, they were asked to exercise continuously for 5 min, performing recumbent cycling at level 8 to measure the HR_max_ (Wallert and Madison, 2014). The participants understood the whole process of the experiment and performed preexperiment training.

### Experimental Design and Process

**Figure 2:**
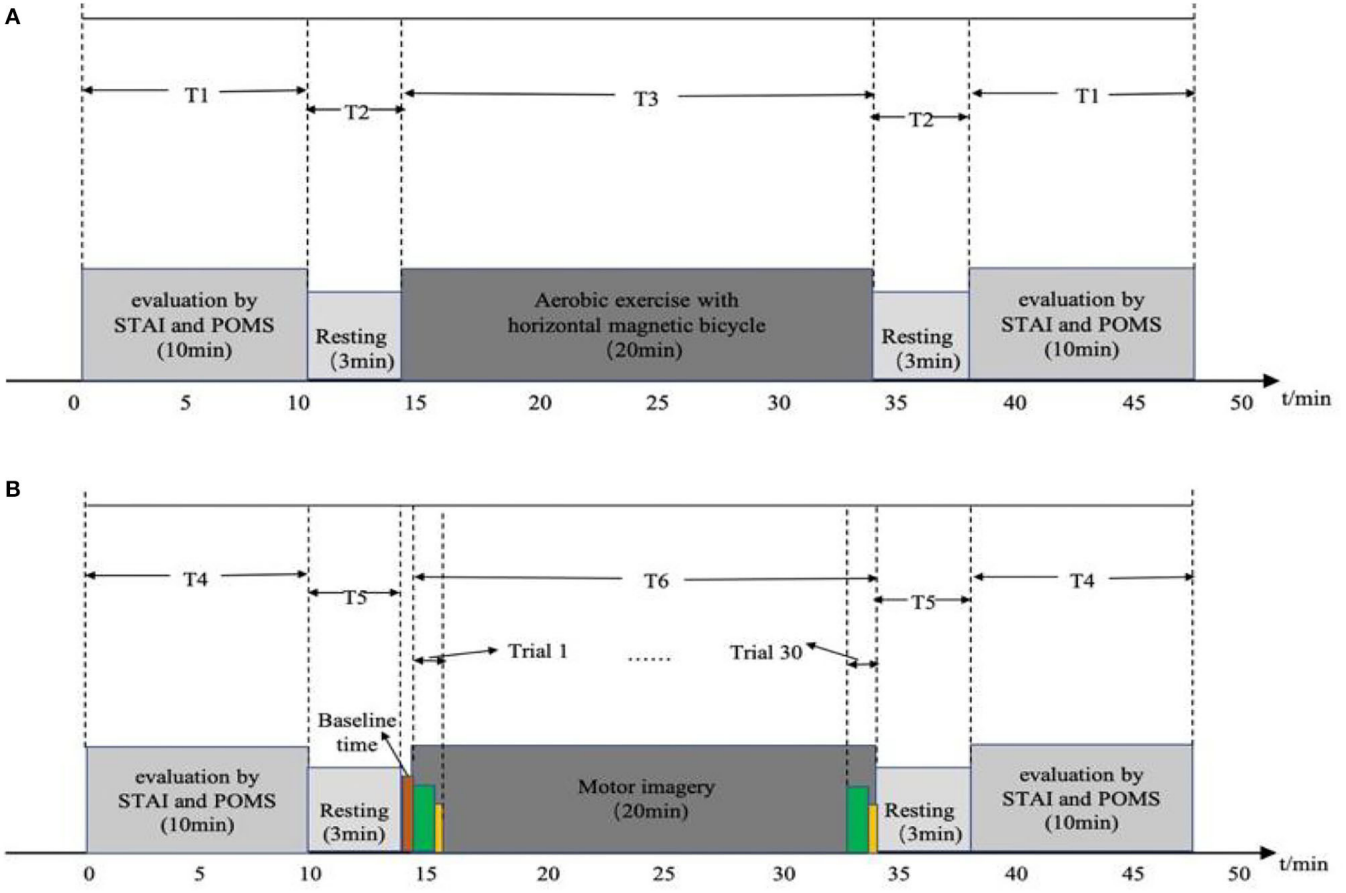
Diagrams of experimental timing: **(A)** timing of aerobic exercise; **(B)** timing of motor imagery.

The diagram for experiment timing is shown in Figure 2. Figure 2A is a time diagram of the experiment of aerobic exercise regulating emotion. During the T1 period, participants filled out the STAI and POMS questionnaires for 10 min; then, a computer voice prompted them to stay awake and relaxed for 3 min, which is the T2 period. At the end of this rest, the voice prompted the emotion regulation by aerobic exercise test to begin and the participant performed aerobic exercise on the horizontal magnetic bicycle for a continued 20 min, which is the T3 time period. Finally, after the end of the aerobic exercise period, the voice prompts the participant to rest for 3 min, then complete the STAI and POMS questionnaires again.

Figure 2B is the timing diagram of the motor imagery experiment. The participants filled in the STAI and POMS questionnaires during the T4 time period for 10 min; then, a computer voice prompted them to stay at rest for 3 min, which is the T5 time period, before beginning the baseline period, in which they were asked to stay awake and relax for 1 min, without performing any mental tasks. At the end of the baseline state, a voice and picture prompted the start of the motor imagery experiment, which lasts for 2 s, before the participant imagines doing aerobic exercise with a horizontal magnetic bicycle for 30 s. During this period, the computer screen was blank. After the imagery task is over, the participant was asked to rest for 10 s; this constitutes the end of a trial. A total of 30 trials, 20 min in length, composed the T6 time period. Then a voice and picture prompted the participant to rest, asking them to stay awake and relaxed for 3 min, and then fill out the STAI and POMS questionnaires again. The timing of the experiment was implemented by MATLAB Psychtoolbox-3 (R2018a; MathWorks, Natick, MA, USA).

### Experimental Equipment and Data Collection

**Figure 3:**
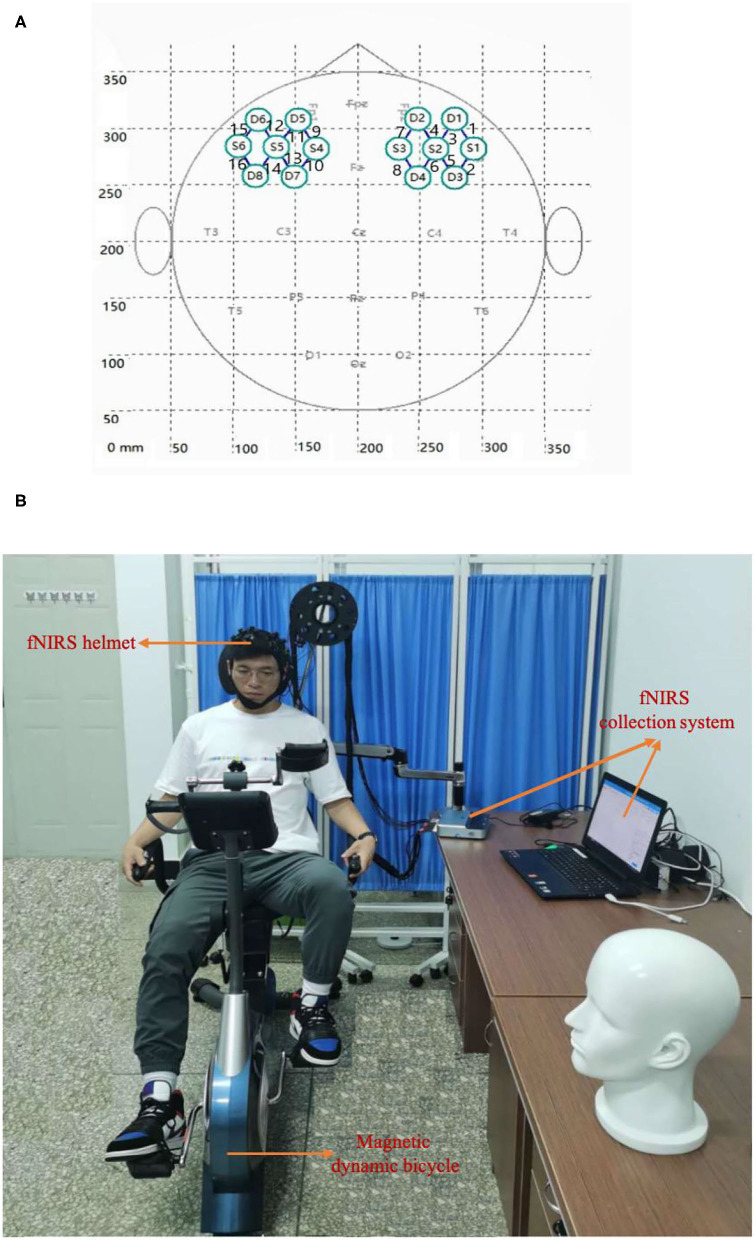
Experimental setup. **(A)** The arrangement of the light source and detector probe. S and D denote the light source probe and the detector probe, respectively. The connecting line between the light source probe and the detector probe denotes the channel, and the number (1–16) denotes the channel identifier. **(B)** Real experiment scene with horizontal magnetic bicycle and fNIRS collection system.

The fNIRS device used in this experiment was a portable Nir Smart [two wavelengths: 760 and 850 nm, 16 channels (eight light sources and eight detectors); Danyang Huichuang Medical Equipment Co., Ltd., Danyang, China]. According to the 10–20 international standard lead system, the fNIRS helmet was placed on the head of the participant such that the light poles covered the left and right prefrontal areas of the brain, including eight channels of each of the left and right prefrontal lobes (3 × 4 array of emitter and detection light poles). The left and right areas were symmetrical and the left and right prefrontal medial channels were located at Fp1 and Fp2, respectively. The emission and detection light poles were arranged as shown in Figure 3A.

The fNIRS sampling rate was 20 Hz, the single wavelength power of the light source was >20 mW, the time resolution was 100 Hz, the dynamic range was >110 dB, and the digital-to-analog conversion accuracy was 24-bit. Data collection was completed according to the experimental timing and requirements in Figure 2A.

The aerobic exercise equipment used in the experiment was a horizontal magnetic bicycle, model JTH-735RS-1 (size: 120^*^50^*^122CM; two 6-KG two-way rotating flywheels, resistance: magnetic control eight-speed resistance adjustment, foot distance: 56–72 CM, load-bearing: 120 kg; Guangzhou Jintong Fitness Equipment Co., Ltd., Guangzhou, China). The equipment had a digital dashboard, which can display parameters such as exercise time, speed, mileage, heart rate, and calories burned. The real experimental scene is shown in Figure 3B. There were 16 channels in total, of which channels one to eight were located in the right prefrontal cortex (PFC) and channels nine to 16 were located in the left PFC.

Before aerobic exercise, the average heart rate of 15 participants in the aerobic exercise group was 75 bpm. Heart rate is the most direct indicator of the effect and intensity of aerobic exercise (the appropriate heart rate for aerobic exercise is 120–135 bpm, as determined by sports medicine) (Ekkekakis et al., 2013). The aerobic exercise in the study was set at a moderate exercise intensity (60–80% of the maximum heart rate). During aerobic exercise, the average heart rate was 128 bpm, the average number of calories burned was 101 kcal, and the average exercise mileage was 3.39 kilometers.

### Data Processing

#### NIRS Data Preprocessing and Feature Extraction

The fNIRS signal collected in the experiment was the original light-intensity signal, which needed to be converted using the improved Lambert-Beer law to discern HbO and HbR concentrations, which are denoted by the relative change values of ΔOxy-Hb and ΔDeoxy-Hb (Cui et al., 2010).

After the fNIRS data were Butterworth band-pass filtered and corrected for baseline drift, the HbO, HbR, and HbT signals were extracted, respectively, and the means of the three signals across all participants and the average concentration changes before and after aerobic exercise, and motor imagery were calculated.

The study also calculated the ratio of HbO concentration in the left and right prefrontal lobes before and after aerobic exercise and motor imagery.

#### Evaluation of STAI and POMS Scores

State–trait anxiety inventory was compiled by Charles Spielberger in 1977 (X version) and revised in 1983 (Y version) (Spielberger et al., 1970; Spielberger, 1983). This scale is characterized by simplicity, high validity, and easy analysis. It can intuitively reflect the participative feelings of anxious individuals, especially the current S-AI with T-AI differentiation. Each item of STAI has four (1–4) grades. The grading standards of S-AI are as follows: 1 = not at all, 2 = some, 3 = moderate, 4 = very obvious, while the grading standard of T-AI are: 1 = almost none, 2 = some, 3 = often, 4 = almost always. Positive emotion items (1, 2, 5, 8, 10, 11, 15, 16, 19, 20, 21, 23, 24, 26, 27, 30, 33, 34, 36, and 39; items are scored with a single superscript ^*^) are reverse scored—, that is, they are rated as four, three, two, and one point(s) in the above order and negative emotions are scored positively. The minimum score of the two scales is 20 points and the maximum is 80 points; the higher the score, the higher the degree of anxiety. The degree of anxiety is divided into four levels: no anxiety (≤ 20 points), mild anxiety (21–39), moderate anxiety (40–59), and severe anxiety (60–80).

Profile of mood states is a scale for the evaluation of positive and negative emotion (Curran et al., 1995), which consists of 40 adjectives (corresponding to 40 items), and is rated from zero (not at all) to four (very) points according to the feelings of the participant (Grove and Prapavessis, 1992; Zhu, 1995). The 40 items of the scale correspond to the scores of seven subscales: tension (*n* = 6 items), anger (*n* = 7 items), fatigue (*n* = 5 items), depression (*n* = 6 items), energy (*n* = 6 items), panic (*n* = 5 items), and self-esteem (*n* = 5 items). The total mood disturbance (TMD) score = (tension score + anger score + fatigue score + depression score + confusion score) – (energy score + emotional score related to self-esteem) + 100 (Andrykowski et al., 1990, 1993). Higher TMD scores indicate that the emotional state of the participants is negative.

An STAI score of 40–59 points indicates that an individual has moderate anxiety, while a POMS score of 110–140 points indicates that an individual is in a negative mood. These two scales were limited to these score intervals to screen the participants who are in line with the experiment.

#### Calculation of Spearman's Correlation Coefficient Between Changes in the Average STAI and POMS Scores Before and After Emotion Regulation and Changes in Blood Oxygen Concentration

To gain a more accurate grasp of the emotion regulation of the study participants before and after aerobic exercise and motor imagination, the change of HbO signal was selected as the judgment standard, and the three stages before, during, and after aerobic exercise and motor imagination were selected to draw a real-time dynamic diagram of HbO.

In this study, Spearman's correlation coefficient (Fieller and Pearson, 1961) was used to measure the dependence of the two variables. The correlation coefficient was defined as Pearson's correlation coefficient. The correlation coefficient is used to calculate the correlation between changes in the average STAI and POMS scores and changes in the HbO, HbR, and HbT concentrations before and after aerobic exercise and motor imagery emotion regulation. In this study, MATLAB_2018a (MathWorks) was used to calculate Spearman's correlation coefficient.

## Results

### STAI and POMS Evaluation and fNIRS Data Used Two-Way ANOVA Results

Statistics in Tables 1, 2 revealed that two-way ANOVA was used to test the group factors (aerobic exercise group and motor imagery group) and intervention factors (pretest and posttest) of the participants, respectively. In the pretest and posttest factor analysis (*p* < 0.01), posttest anxiety was significantly lower than pretest anxiety. Among the groups (*p* < 0.01), the anxiety degree of the aerobic group was significantly lower than that of the motor imagery group. The laterality ratio was the left and right PFC fNIRS concentration ratio, similar to the laterality score gleaned when using EEG to assess PFC asymmetry (Palmiero and Piccardi, 2017).

**Table 1:** Changes in STAI and POMS scores before and after aerobic exercise and motor imagery^*^.

	**Pre-AE**	**Post-AE**	***F***	***p***	**Pre-MI**	**Post-MI**	***F***	***p***
STAI (M ± SD)	43.27 ± 2.49	36 ± 1.90	75.47	*p < * 0.01	43.87 ± 5.67	42.87 ± 5.80	0.80	0.38
POMS (M ± SD)	123.67 ± 4.54	96.67 ± 3.52	309.48	*p < * 0.01	123.40 ± 4.63	121.73 ± 3.70	1.11	0.30

* *Two-way ANOVA test showed significant results*.

*AE, aerobic exercise; M ± SD, mean ± standard deviation; MI, motor imagery; POMS, Profile of Mood States; STAI, State-Trait Anxiety Inventory*.

**Table 2:** Mean and standard deviation values of HbO concentration changes in the left and right prefrontal areas before and after aerobic exercise and motor imagery^*^.

	**Pre (M ± SD)**	**Post (M ± SD)**	**Degrees of freedom**	***F***	***P***
**Aerobic exercise group**					
Left prefrontal cortex	−0.032 ± 0.308	0.100 ± 1.025	1.8	25.67	*p < * 0.01
Right prefrontal cortex	−0.048 ± 0.373	−0.142 ± 1.089		5.16	0.04
HbO activation concentration ratio	1.404				
**Motor imagery group**					
Left prefrontal cortex	−0.039 ± 0.365	0.016 ± 0.391	1.8	4.5	0.06
Right prefrontal cortex	−0.034 ± 0.379	−0.011 ± 0.405		3.7	0.07
HbO activation concentration ratio	2.391				

* *Two-way ANOVA test showed significant results*.

*M ± SD, mean ± standard deviation*.

Table 3 presents the mean and standard deviation values of the HbO, HbR, and HbT concentration changes in the prefrontal area between before and after aerobic exercise and motor imagery. The average changes in the concentrations of HbO, HbR, and HbT in the prefrontal lobe area increased after aerobic exercise; meanwhile, considering the prefrontal lobe area after motor imagery, the average changes in HbO, HbR, and HbT concentrations also increased to a certain extent, but the range was small.

**Table 3:** The mean and standard deviation of HbO, HbR, and HbT concentration changes in the prefrontal area before and after aerobic exercise and motor imagery.

	**Aerobic exercise group**			**Motor imagery group**		
	**HbO**	**HbR**	**HbT**	**HbO**	**HbR**	**HbT**
Before aerobic exercise	−0.039	−0.03	−0.01	−0.036	−0.002	−0.013
or motor imagery, M ± SD	± 1.141	± 0.562	± 1.643	± 0.414	± 0.134	± 0.26
After aerobic exercise	0.121	0.047	0.168	0.013	0.002	−0.012
or motor imagery, M ± SD	± 1.058	± 0.514	± 1.502	± 0.398	± 0.136	± 0.266

*M ± SD, mean ± standard deviation*.

### Results of Spearman's Correlation Coefficient Analysis

Table 4 presents the Spearman correlation coefficients calculated using the average changes of the STAI and POMS scores between before and after aerobic exercise and the changes in HbO, HbR, and HbT concentrations. The results showed that the average STAI and POMS scores before and after aerobic exercise were negatively correlated with the concentrations of HbO, HbR, and HbT.

**Table 4:** Spearman correlation coefficients of changes in the average scores of STAI and POMS scales before and after aerobic exercise and changes in HbO, HbR, and HbT concentrations.

	**STAI and POMS scores**		**Aerobic exercise group**		
	**STAI**	**POMS**	**HbO**	**HbR**	**HbT**
Before aerobic exercise	43.27 ± 2.49	123.67 ± 4.54	−0.039 ± 1.141	−0.03 ± 0.562	−0.01 ± 1.643
After aerobic exercise	36 ± 1.90	96.67 ± 3.52	0.121 ± 1.058	0.047 ± 0.514	0.168 ± 1.502
Spearman's correlation coefficient calculation			−1	−1	−1

### Change in HbO Concentration

**Figure 4:**
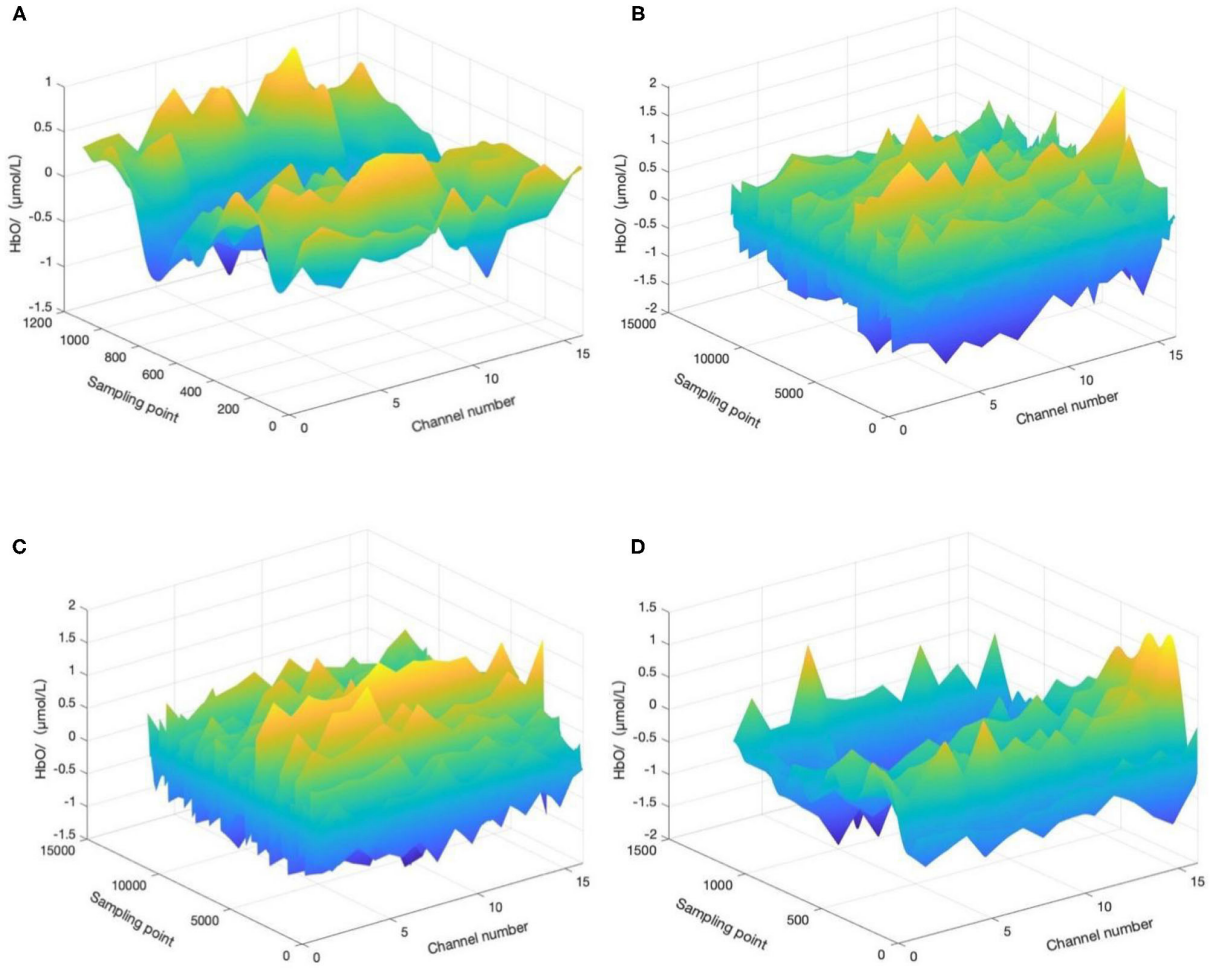
HbO concentration changes before, during, and after aerobic exercise. **(A)** HbO concentration change during −1 to 0 min, **(B)** HbO concentration change during 0–10 min, **(C)** HbO concentration change during 10–20 min, and **(D)** HbO concentration change during 20–21 min. *T* = 0 corresponds to the start time of aerobic exercise.

For the convenience of discussion, the start time of aerobic exercise was specified as *t* = 0 min (*t* = −1 means 1 min before aerobic exercise, *t* = 10 means 10 min after the start of aerobic exercise, *t* = 20 means the end time of aerobic exercise, and *t* = 21 means 1 min after the end of aerobic exercise). We selected four time periods (−1 to 0 min, 0–10 min, 10–20 min, and 20–21 min) and calculated and the HbO concentration change in the corresponding period, as shown in Figures 4A–D.

A minute before the start of aerobic exercise, the change in HbO concentration tends to be flat, without a prominent peak signal, and the participant may still be in a state of depression and anxiety. Ten minutes after the start of aerobic exercise, the HbO concentration has a peak signal, with a largely positive change, and the depression and anxiety mood of the patient may transform into a positive mood. Twenty minutes after the start of aerobic exercise, the positive change in HbO concentration has increased compared relative to during the previous 10 min, and the depression and anxiety mood of the participant may continue to transform into a positive mood. Finally, 1 min after the end of aerobic exercise, the HbO concentration evolves to its peak, and the effect of aerobic exercise on improving negative emotion continues.

**Figure 5:**
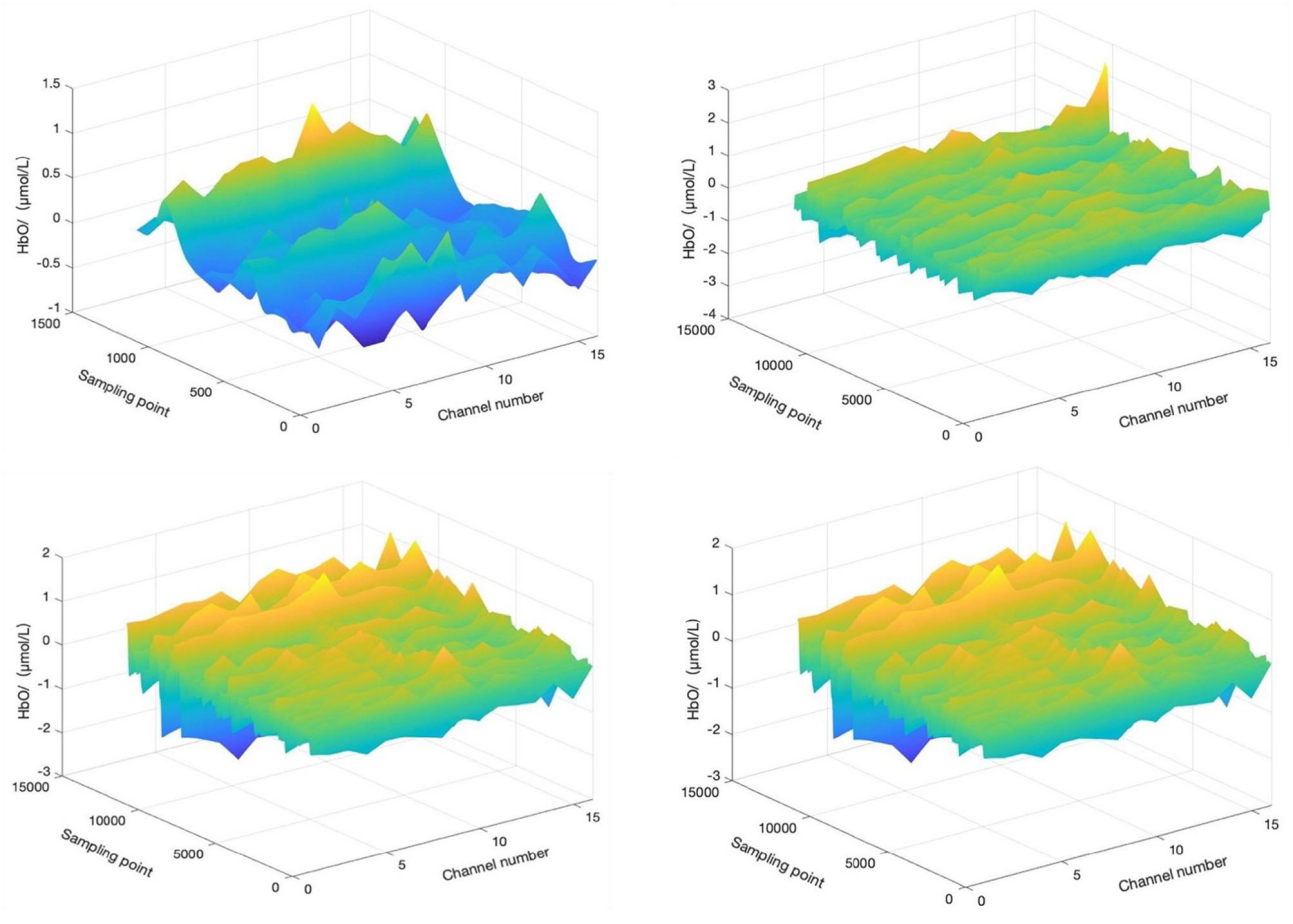
HbO concentration changes before, during, and after motor imagery. The top left is HbO concentration change during −1 to 0 min. The top right is HbO concentration change during 0–10 min. The bottom left is HbO concentration change during 10–20 min, and the bottom right is HbO concentration change during 20–21 min. *T* = 0 corresponds to the start time of motor imagery.

For the convenience of discussion, the start time of motion imagery was specified as *t* = 0 min (*t* = −1 means 1 min before motion imagery, *t* = 10 means 10 min after the start of aerobic exercise, *t* = 20 means the end time of aerobic exercise, and *t* = 21 means 1 min after the end of aerobic exercise). We selected four time periods (−1 to 0 min, 0–10 min, 10–20 min, and 20–21 min) and calculated and the HbO concentration change in the corresponding period, as shown in Figure 5.

A minute before the start of the motor imagery, the HbO concentration change tends to be flat, without a prominent peak signal, and the mood of the participant may still be in a state of depression and anxiety. Ten minutes after the start of the motor imagery, the HbO concentration changes little, yet the overall trend is still flat, although depression and anxiety may show a small conversion to positive emotion. Twenty minutes after the start of motor imagery, the HbO concentration changes little relative to in the first 10 min and still tends to be flat. The depression and anxiety of the participants may still experience a small conversion to positive emotion. Finally, 1 min after the end of motor imagery, the HbO concentration changes with a small peak, and the effect of motor imagery on improving negative emotion continue, but the improvement may be small.

## Discussion

To further verify the above results, ANOVA was used to analyze the changes in STAI and POMS scores before and after aerobic exercise and motor imagery. The results showed that the scores of the two scales changed significantly between before and after emotion regulation by aerobic exercise, and depression and anxiety emotions transformed into positive emotions. Before and after motor imagery emotion regulation, the scores of the two scales changed, but not significantly, and the transformation of depression and anxiety into positive emotion was not significant.

To reveal the relationship between changes in the blood oxygen concentration (Hb) and changes in the STAI and POMS scores, Spearman's correlation coefficient analysis revealed that the changes in the average STAI and POMS scores before and after aerobic exercise and motor imagery emotion regulated the changes in HbO, HbR, and HbT concentrations in a negatively correlated manner, which indicates that the decrease in average STAI and POMS scores before and after aerobic exercise and motor imagery emotion regulation decrease (due to the conversion of negative emotion to positive emotion) corresponds to an increase in HbO, HbR, and HbT concentrations.

Table 5 shows the comparison between this study and other related research. Bernstein et al. (Bernstein and Mcnally, 2018) concluded that, as compared with a stretching exercise group, a bicycle exercise group attained a more significant effect on emotion regulation. Meanwhile, Subramaniapillai et al. (Subramaniapillai et al., 2016) reported that adolescents with bipolar disorder would also feel the positive emotional benefits brought about by exercise. Szabo et al. (2015) concluded that, after aerobic exercise, positive emotions increased, and negative emotions decreased. Knapen et al. (2009) showed that the state of anxiety and negative emotions are not affected by aerobic exercise type. When study participants chose their exercise intensity, they influenced the happiness and fatigue changes brought about by exercise in positive and negative ways. The results of Zhang et al. (2018) showed that short-term aerobic exercise significantly improved the executive function and emotional regulation ability of female college students with anxiety, where the lower the level of aerobic adaptability, the better the improvement effect of short-term aerobic exercise on unconscious cognition.

**Table 5:** Comparison of this and other related studies.

**Study**	**Exercise manner**	**Scale**	**Features extracted**	**Analytical method(s)**
Bernstein and Mcnally (2018)	Bicycle exercise Stretching exercises	SES DAS	Heart rate SES score DAS score	Independent-samples *t*-test, linear regression, maximum likelihood estimation
Subramaniapillai et al. (2016)	Bicycle Dynamometer	EFI RPE	EFI score RPE score	Independent-samples *t*-test
Szabo et al. (2015)	Exercise bicycle	PANAS	Heart rate PANAS mean and standard deviation values	Wilcoxon signed-rank test
Knapen et al. (2009)	Electronic brake bicycle force gauge	STAI SEES	Heart rate STAI score SEES score	SAS program, mixed repeated measurement variance analysis
Zhang et al. (2018)	International affective picture system, power bicycle	PANAS SAS	PANAS score SAS score	Independent-samples t-test, repeated measures ANOVA
This study	Horizontal magnetic bicycle, motor imagery	STAI POMS	Heart rate STAI score POMS score Before and after aerobic exercise and motor imagery; HbO, HbR, and HbT mean concentrations	ANOVA, Spearman's correlation coefficient, fNIRS analysis

Compared with the above-mentioned studies, the present study uses relatively new fNIRS brain function imaging technology to quantitatively monitor and evaluate changes in brain tissue blood oxygen concentrations (HbO, HbR, and HbT) during emotion regulation by aerobic exercise and motor imagery. Here, participants' emotions were regulated by horizontal magnetic bicycle and motor imagination, and STAI and POMS scores were used to evaluate the emotional changes in participants before and after aerobic exercise and motor imagery. The results showed that, during the period of aerobic exercise emotion regulation, HbO can represent the metabolic activity of oxyhemoglobin in brain tissue and indirectly reflect the activity of the neuron group according to the neurovascular coupling relationship. Previous studies have found that when individuals evolve from negative emotion to positive emotion, the concentration of HbO in the left PFC increases, while that in the right PFC decreases (Ochsner et al., 2004; Kim and Hamann, 2007). Two-way ANOVA was used to test the group factors (aerobic exercise group and motor imagery group) and intervention factors (pretest and posttest) of the participants, respectively. Considering the pretest and posttest factors (*p* < 0.01), posttest anxiety was significantly lower than pretest anxiety. Considering group factors (*p* < 0.01), the anxiety degree of the aerobic group was significantly lower than that of the motor imagery group. The increase in the left PFC HbO concentration and functional activation and the decrease in the right PFC HbO concentration and functional deactivation may be related to the reduction in STAI and POMS scores. Furthermore, Spearman's correlation coefficient of the STAI and POMS scores and HbO concentration were calculated, and the result was −1, indicating that they were completely negatively correlated.

The above results show that fNIRS can be effectively applied to the monitoring and evaluation of emotion regulation by aerobic exercise. In comparison with aerobic exercise, motor imagery has no significant effect on state-trait anxiety and mood state, but it may play an auxiliary role in regulating emotion.

The disadvantage of this study is that single-mode fNIRS brain imaging technology records single brain activity, and its spatial and temporal resolutions are limited. Emotion regulation is a medium- and long-term change process, but this study involved just 20 min of aerobic exercise. Future research should incorporate long-term tracking of aerobic exercise to regulate emotion.

## Conclusion

This study showed that the portable fNIRS could be effectively used for monitoring and evaluating emotion regulation by aerobic exercise on a horizontal magnetic bicycle. The effects of aerobic exercise on emotion regulation were more significant than those of motor imagery, and the effect of motor imagery on emotion regulation was limited, although carrying out motor imagery on the basis of aerobic exercise may be beneficial to enhance the effect of emotion regulation. This study was expected to provide ideas for constructing fNIRS-based online real-time monitoring and the evaluation of emotion regulation by aerobic exercise and motor imagery, which could be used to monitor and evaluate individual state–trait anxiety and mood states.

## Data Availability Statement

The original contributions presented in the study are included in the article/supplementary material, further inquiries can be directed to the corresponding author/s.

## Ethics Statement

The studies involving human participants were reviewed and approved by Medical Ethics Committee of Kunming University of Science and Technology School of Medicine. The patients/participants provided their written informed consent to participate in this study. Written informed consent was obtained from the individual(s) for the publication of any potentially identifiable images or data included in this article.

## Author Contributions

All authors listed have made a substantial, direct and intellectual contribution to the work, and approved it for publication.

## Funding

This work was supported by Fund projects: NSFC (81771926, 61763022, 81470084, 61463024, 62006246, and 32060196).

## Conflict of Interest

The authors declare that the research was conducted in the absence of any commercial or financial relationships that could be construed as a potential conflict of interest.

## Publisher's Note

All claims expressed in this article are solely those of the authors and do not necessarily represent those of their affiliated organizations, or those of the publisher, the editors and the reviewers. Any product that may be evaluated in this article, or claim that may be made by its manufacturer, is not guaranteed or endorsed by the publisher.

## Appendix

**Table:** Changes of HBO concentration in each channel before and after aerobic exercise and motor imagery.

**Channel number**	**1**	**2**	**3**	**4**	**5**	**6**	**7**	**8**
Pre-AE	0.003 ± 0.39	−0.016 ± 0.25	−0.014 ± 0.34	−0.043 ± 0.32	−0.052 ± 0.32	−0.096 ± 0.45	−0.042 ± 0.34	−0.119 ± 0.50
Post–AE	0.067 ± 0.37	0.171 ± 0.33	0.129 ± 0.43	0.210 ± 0.41	0.337 ± 0.39	0.055 ± 0.32	0.115 ± 0.36	0.050 ± 0.42
Pre–MI	−0.036 ± 0.32	−0.034 ± 0.36	−0.044 ± 0.33	−0.039 ± 0.35	−0.033 ± 0.39	−0.024 ± 0.35	−0.022 ± 0.38	−0.040 ± 0.41
Post-MI	0.018 ± 0.29	0.005 ± 0.19	−0.007 ± 0.28	0.017 ± 0.31	0.013 ± 0.35	0.046 ± 0.27	−0.004 ± 0.24	−0.003 ± 0.21
**Channel number**	**9**	**10**	**11**	**12**	**13**	**14**	**15**	**16**
Pre–AE	−0.031 ± 0.42	−0.087 ± 0.28	−0.062 ± 0.32	−0.023 ± 0.37	−0.058 ± 0.28	−0.023 ± 0.23	0.007 ± 0.31	0.025 ± 0.17
Post–AE	0.156 ± 0.45	0.127 ± 0.38	0.324 ± 0.33	0.071 ± 0.51	0.171 ± 0.43	0.021 ± 0.33	0.182 ± 0.35	0.210 ± 0.31
Pre–MI	−0.035 ± 0.42	−0.008 ± 0.33	−0.039 ± 0.36	−0.063 ± 0.32	−0.027 ± 0.36	−0.036 ± 0.32	−0.066 ± 0.35	−0.036 ± 0.43
Post-MI	0.010 ± 0.36	−0.012 ± 0.34	0.020 ± 0.28	0.040 ± 0.23	−0.005 ± 0.37	0.011 ± 0.32	0.051 ± 0.29	0.013 ± 0.22
